# Off-pump repair of a post-infarct ventricular septal defect: the 'Hamburger procedure'

**DOI:** 10.1186/1749-8090-1-10

**Published:** 2006-05-12

**Authors:** Thomas A Barker, Alexander Ng, Ian S Morgan

**Affiliations:** 1 Department of Cardiothoracic Surgery, Heart and Lung Centre, Wolverhampton, UK; 2 Department of Cardiothoracic Anaesthesia, Critical Care & Pain Management, Heart and Lung Centre, Wolverhampton, UK

## Abstract

We report a novel off-pump technique for the surgical closure of post-infarct ventricular septal defects (VSDs). The case report describes the peri-operative management of a 76 year old lady who underwent the 'Hamburger procedure' for closure of her apical VSD. Refractory cardiogenic shock meant that traditional patch repairs requiring cardiopulmonary bypass would be poorly tolerated. We show that echocardiography guided off-pump posterior-anterior septal plication is a safe, effective method for closing post-infarct VSDs in unstable patients. More experience is required to ascertain whether this technique will become an accepted alternative to patch repairs.

## Introduction

Ventricular septal defect (VSD) is a rare but significant complication following myocardial infarction. Medical management alone is inadequate, and although recent advances in transcatheter closure have been promising [1], surgical repair if often the only option. Various surgical techniques have been described including single or double patch procedures with infarct exclusion [2,3]. These open procedures require the use of cardiopulmonary bypass (CPB) and 30-day mortality ranges from 23 to 42% [3,4].

Recently, an off-pump closure technique called the 'Hamburger procedure' has been pioneered as an alternative to open procedures that require CPB and ventriculotomy [5]. The aim of our report is to describe how a post-infarct VSD may be repaired without CPB and to highlight the importance of echocardiography to guide VSD closure.

## Case report

A 76 year-old female smoker was thrombolyzed with Tenecteplase after an acute anterior myocardial infarction. Unfortunately she remained hypotensive and so she underwent emergency angiography which showed patent coronary arteries except for the left anterior descending artery which was occluded distally. After this procedure she was noted to have a pansystolic murmur and transthoracic echocardiography (TTE) using a Vivid 7 Pro (GE Vingned, Norway), confirmed a 1 cm antero-apical VSD and an akinetic ventricular apex.

To manage her hypotension inotropes were commenced and an intra-aortic balloon pump was inserted. After stabilization for 5 days, diuretics and nitrates were added for progressing pulmonary oedema. Repeat TTE images showed no change, and although the right ventricle was mildly dilated, global left ventricular function was preserved. A decision was made to perform a delayed surgical repair to reduce operative risk.

She was reviewed in the local multi-disciplinary meeting and transcatheter closure was not thought to be appropriate. Four weeks after admission, she developed short episodes of ventricular tachycardia which were controlled by amiodarone. Two days later she deteriorated becoming hypotensive with worsening pulmonary oedema. She became hypoxic and hypercarbic and dobutamine infusion was added along with an escalation in her diuretic therapy. Sedation and mechanical ventilation were then required. Repeat TTE remained unchanged. A pulmonary artery flotation catheter was inserted and norepinephrine was commenced to treat persistent hypotension. Renal function began to deteriorate with serum creatinine rising to 190 μmol l^-1^.

**Figure 1:**
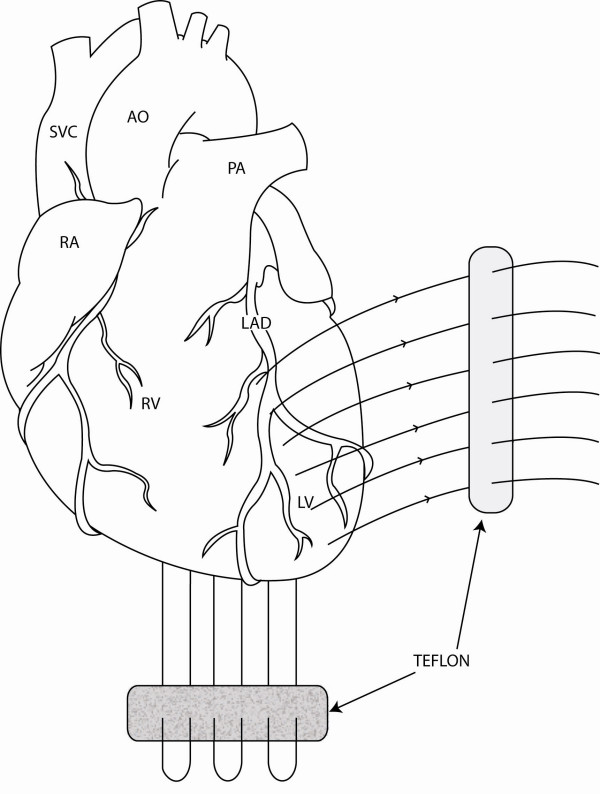
Diagram of the heart demonstrating suture position for the 'Hamburger procedure'. AO = aorta, LAD = left anterior descending artery, LV = left ventricle, PA = pulmonary artery, RA = right atrium, RV = right ventricle, SVC = superior vena cava.

**Figure 2:**
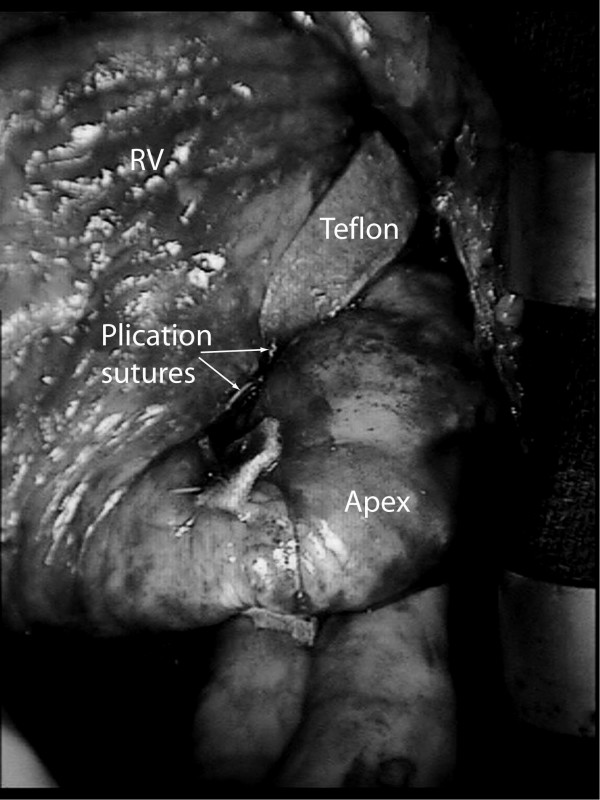
Operative photograph of the heart following the 'Hamburger procedure'.

**Figure 3:**
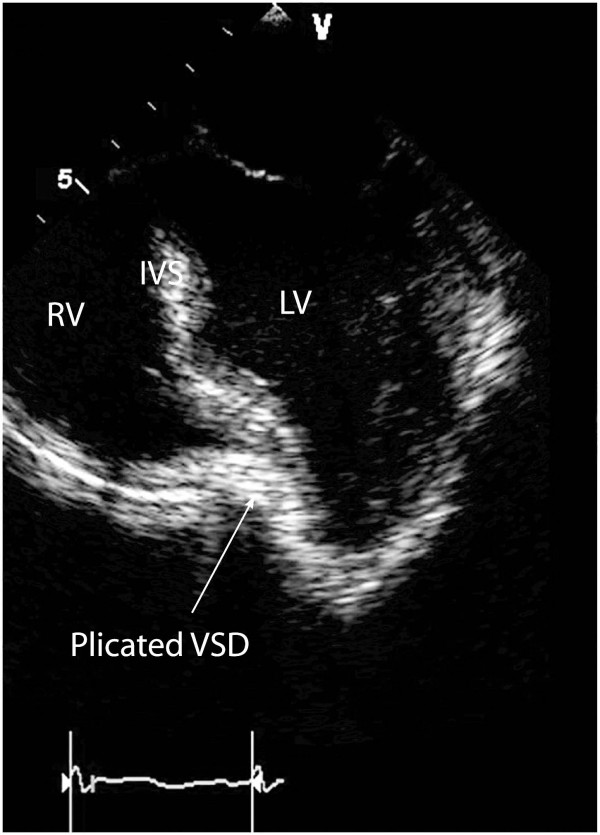
Mid-esophageal, 4 chamber, 2D Transoeophageal echocardiogram showing septal plication. IVS = interventricular septum.

Thirty days after myocardial infarction, she underwent an off-pump 'Hamburger' post-infarct VSD repair. The heart was approached through a median sternotomy and a posterior-anterior septal plication was performed using three double-armed Teflon felt supported interrupted 1.0 Ticron sutures (Syneture™, USA). The Teflon strip was preloaded with sutures and from below the needles were passed through the posterior (inferior) interventricular septum aiming for the anterior part of the septum where the tip of the needle is retrieved. The sutures run just lateral to the LAD to ensure plicating the thicker left ventricular wall rather than the thinner right ventricular wall (Figure 1). The needles were then passed through the second Teflon strip and then tied starting at the apex and working more proximally (Figure 2). VSD closure was assessed using transesophageal (Figure 3) and epicardial echocardiography as well and by epicardial auscultation.

Soon after surgery, she began to improve and her inotropes and frusemide were discontinued. However she required a tracheotomy to facilitate weaning from mechanical ventilation. She was transferred to a rehabilitation facility 4 weeks after the operation. She was found to be asymptomatic and mobilising independently at 6 month follow-up.

## Comment

Here we show that the 'Hamburger procedure' was an effective method for repair of a post-infarct VSD. By bringing the left and right ventricles into close apposition with Teflon supported plication sutures, the defect was closed with moderate reductions in cardiac chamber size. A dramatic reduction in pulmonary arterial pressure and inotropic doses occurred immediately after surgery. As our technique was performed off-pump, potential sequelae of CPB (ie adverse haemodynamic, neurological and inflammatory effects) were obviated. This lady benefited from a short operative time of 40 minutes compared with a longer, more complex open patch repair requiring CPB and ventriculotomy.

One important factor associated with poor outcome is a short VSD to operation time [6]. Surgery was postponed for 4 weeks which allowed the fibrotic process in the infarcted myocardium to become established. This delay helped in allowing the Teflon supported plication sutures to hold. It is uncertain whether the friable myocardium would have supported such sutures if surgery was performed sooner.

We demonstrate that echocardiography is not only essential in the diagnosis of post-infarct VSDs but also plays an important role in guiding VSD closure when the 'Hamburger procedure' is utilized. In this case, extra sutures were added to the initial plication after echocardiography had demonstrated that VSD closure was incomplete. Both transoesophageal and epicardial imaging enabled us to ensure that successful closure was achieved. TTE was used in the post-operative period to show that there was no residual VSD prior to discharge.

The 'Hamburger procedure' is a promising alternative method for post-infarct VSD repair. It appears to be suitable in patients with a VSD in the antero-apical septal position. We feel this technique would not be appropriate for posterior VSDs or VSDs higher up the septum closer to the atrioventricular valves. A randomised controlled trial would be needed to compare outcomes after this technique with those after conventional methods. VSD recurrence rates, functional outcome and mortality would be important factors that could be assessed. In this case, we believe that the risk of morbidity and mortality was reduced by avoiding CPB and therefore we are confident that the 'Hamburger Procedure' may become an accepted and useful technique in the future.
